# Identification of Indicators for Preterm Birth Using Retinoid Metabolites

**DOI:** 10.3390/metabo11070443

**Published:** 2021-07-07

**Authors:** Young-Ah You, Soo-Yeon Hwang, Soo-Min Kim, Seojeong Park, Ga-In Lee, Sunwha Park, AbuZar Ansari, Jeongae Lee, Youngjoo Kwon, Young-Ju Kim

**Affiliations:** 1 Department of Obstetrics and Gynecology, Ewha Medical Institute and College of Medicine, Ewha Womans University, Seoul 07804, Korea; yayou@ewha.ac.kr (Y.-A.Y.); clarrissa15@gmail.com (S.P.); abu.kim.0313@gmail.com (A.A.); 2 Department of Pharmacy, Ewha Womans University, Seoul 03760, Korea; syhwang428@ewha.ac.kr (S.-Y.H.); seoj_park@ewha.ac.kr (S.P.); 3 Graduate Program in System Health Science and Engineering, Ewha Womans University, Seoul 03760, Korea; salz.soom@gmail.com (S.-M.K.); loveleee0102@gmail.com (G.-I.L.); 4 MolecularRecognition Research Center, Korea Institute of Science and Technology, Seoul 02792, Korea; frans@kist.re.kr

**Keywords:** preterm birth, metabolomic analysis, retinoid metabolism, parturition, 13-cis-retinoic acid

## Abstract

Metabolites reflect the biochemical dynamics for the maintenance of pregnancy and parturition. UPLC-Q/TOF-MS and LC-MS/MS metabolomics were performed to identify and validate the plasma metabolomic signatures of preterm birth (PTB). We recruited pregnant women between 16 and 40 weeks 5 days gestational age at Ewha Womans Mokdong Hospital for a nested case-control study. In untargeted UPLC-Q/TOF-MS, score plots of partial least-squares discriminant analysis clearly separated the PTB group from the term birth (TB, *n* = 10; PTB, *n* = 11). Fifteen metabolites were significantly different between the two groups, as indicated by a variable importance in projection >1 and *p* < 0.05. Metabolic pathways involving retinol, linoleic acid, d-arginine, and d-ornithine were associated with PTB. Verification by LC-MS/MS focused on retinol metabolism (TB, *n* = 39; PTB, *n* = 20). Retinol levels were significantly reduced in PTB compared to TB, while retinal palmitate, all-trans-retinal, and 13-cis-retinoic acid (^13^cis-RA) significantly increased (*p* < 0.05). Retinol-binding protein levels were also elevated in PTB. Additionally, all-trans-retinal (AUC 0.808, 95% CI: 0.683–0.933) and ^13^cis-RA (AUC 0.826, 95% CI: 0.723–0.930) showed improved predictions for PTB-related retinol metabolites. This study suggests that retinoid metabolism improves the accuracy of PTB predictions and plays an important role in maintaining pregnancy and inducing early parturition.

## 1. Introduction

Preterm birth (PTB) is defined as giving birth before 37 weeks of gestation and is a major cause of death in children below 5 years worldwide [1]. Additionally, complications of PTB among some survivors may affect long-term life, impairing neurodevelopmental functioning by increasing the risk of cerebral palsy, causing learning impairments and visual disorders, and affecting long-term physical health with a higher disease risk [2]. Although the potential cause of PTB includes a wide range of pathological processes associated with risk factors, including biological, environmental, behavioral, and social influences and interactions between maternal and fetal factors, the main causes of PTB is unclear and often complex [3,4,5,6,7,8,9].

Clinical methods to predict preterm birth include a history of preterm birth, cervical length (CL) in the 2nd trimester, cervical elastography, and biochemical markers. Although fetal fibronectin (fFN) is commercially available in the clinical field, its sensitivity is only 56% and has low prediction rate; even in asymptomatic women, although specificity is 97%, the sensitivity is 26% [10,11]. Improving preterm morbidity and mortality therefore requires a greater understanding of the processes associated with PTB and the biomarkers that can accurately predict PTB.

Spontaneous PTB results from preterm labor and preterm premature rupture of membrane (pPROM) induced by various pathological processes in approximately 70% of cases [12,13]. It is not well known how the process of PTB is initiated, but the main pathways are fetal and maternal tissue activation by cervical insufficiency, stress, inflammation, hemorrhage, uterine distension, and immune dysregulation. These risks result in myometrium contractions and/or rupture of the fetal membranes by the release of mediators, including prostaglandins and interleukins (ILs) [14]. Also, membrane rupture is related to biochemical changes in collagen structure and formation, as well as increased oxidative stress [15,16], involving an imbalance between synthesis and matrix metalloproteinase-induced collagen degradation within the extracellular matrix of the chorioamniotic membrane [17].

Using these pathophysiological pathways, many researchers have sought to identify biomarkers of inflammation related to ascending intrauterine infection to predict PTB [18]. Notably, ascending infection and PTB have been known to be associated with dysbiosis of the vaginal microbiome induced by a shift of dominant species from *Lactobacillus* to *Bacteroides*, *Prevotella*, *Klebsiella*, and *Mobiluncus* [13,19,20,21]. We have also previously reported that increased cytokine levels (MIP-1α, IL-6, and IL-17α) and metabolites (glycolate, ethylene glycol, isopropanol, methanol, formate, and trimethylamine N-oxide) are critically related to PTB [22,23]. Thus, vaginal microbiome dysbiosis and alterations in cytokine and metabolite levels are likely to be predictive biomarkers of ascending intrauterine infection-induced PTB, although further investigations are required.

Studies have reported that PTB-induced oral pathogens can be transmitted to gestational tissues via hematogenous spread [24,25]. Additionally, we previously reported that altered composition of the blood microbiome could affect gestational tissue, leading to PTB [26]. Changes in blood microbiome can affect metabolite production, which can lead to outbreaks of various diseases, including PTB [27,28]. Accordingly, we performed metabolome profile analysis in maternal plasma and found that metabolic pathways related to retinol, linoleic acid, d-arginine, and d-ornithine are associated with PTB.

Adequate levels of vitamin A during pregnancy are of critical importance for the health of pregnant women and their fetuses [29]. Low levels of vitamin A may be associated with complications in pregnancy, including death, increased infections, or low iron levels in the mother or baby, or having a baby with any of the following complications: early delivery, low birth weight, or a congenital abnormality [29,30,31]. In higher concentrations, retinoids inhibit cell growth and can be pro-oxidant, cytotoxic, mutagenic, and teratogenic [30,32]. These observations point to the need to reevaluate the role of vitamin A in maternal and child health and in preterm birth in particular [30]. Here, we report the results of validation focused on retinoid metabolism to improve the prediction of PTB and understand its broader mechanism.

## 2. Results

### 2.1. Clinical Charateristics of Subjects

**Figure 1:**
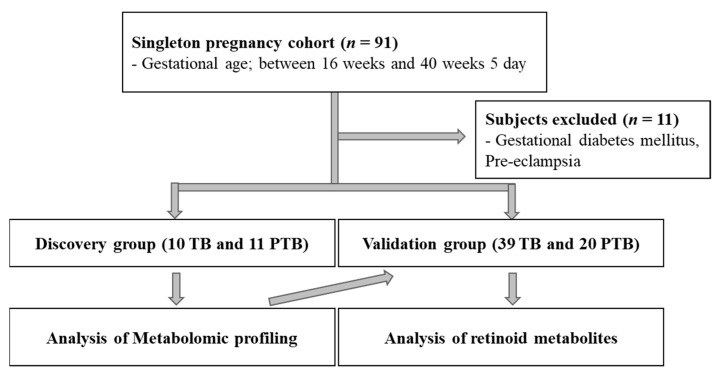
Flowchart for subject selection. TB, term birth; PTB, preterm birth.

We collected peripheral blood from pregnant women, at 16 to 40 weeks and 5 days gestational age to identify the metabolomic signatures of PTB (term birth, TB *n* = 10; PTB, *n* = 11; Table S1) and to validate the targeted metabolites (TB, *n* = 39; PTB, *n* = 20) (Figure 1). Clinical parameters, including gestational age at sampling and delivery were significantly different between the two groups in both the discovery and validation groups (*p* < 0.05, Table 1). The white blood cell count and C-reactive protein levels were also significantly different between the two groups in the validation group (*p* < 0.05). Liver function enzymes, such as alanine and aspartate aminotransferases, and lipid profiles in pregnant women in their 3rd trimester did not show statistically significant differences between the two groups (*p* < 0.05, Table S2). Two PTB patients were identified to have chorioamnionitis, but not women who delivered at term.

### 2.2. The Metabolome Profiling of Maternal Plasma Samples

**Figure 2:**
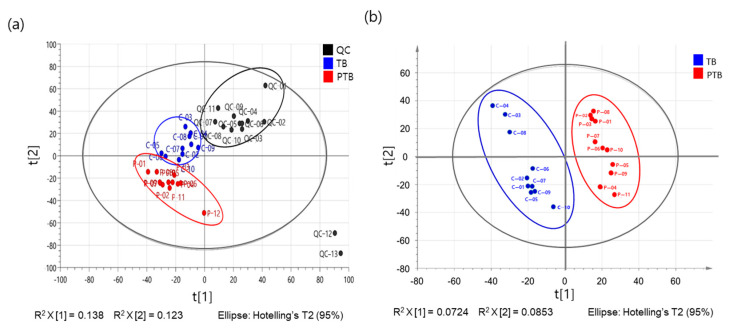
PLS-DA score plots showing that metabolites are differentially categorized in the plasma of PTB compared to TB subjects. (**a**) Quality control (QC) samples (**b**) RPC positive.

To discover PTB-specific metabolites and pathways, a non-targeted metabolite profiling was conducted using ultra-high performance liquid chromatography (UPLC) coupled with an LTQ-Orbitrap Velos Pro hybrid mass spectrometer. Multivariate analysis was performed to obtain a list of variables from variant metabolic profiles in the plasma of pregnant women with TB and PTB. The score plots of partial least-squares discriminant analysis (PLS-DA) were applied to identify metabolomic patterns that could be used to distinguish PTB from the TB group. Figure 2 shows clearly separated categories to PTB compared with TB in reverse phased chromatography (RPC) positive mode (Q^2^ = 0.072; R^2^ = 0.085). Out of 235 variable ions, 15 metabolites were determined based on the value of a variable importance in projection (VIP) larger than 1, and *p*-value less than 0.05. Detailed information is provided in Table S3.

### 2.3. The PTB Related with Metabolic Pathways

**Figure 3:**
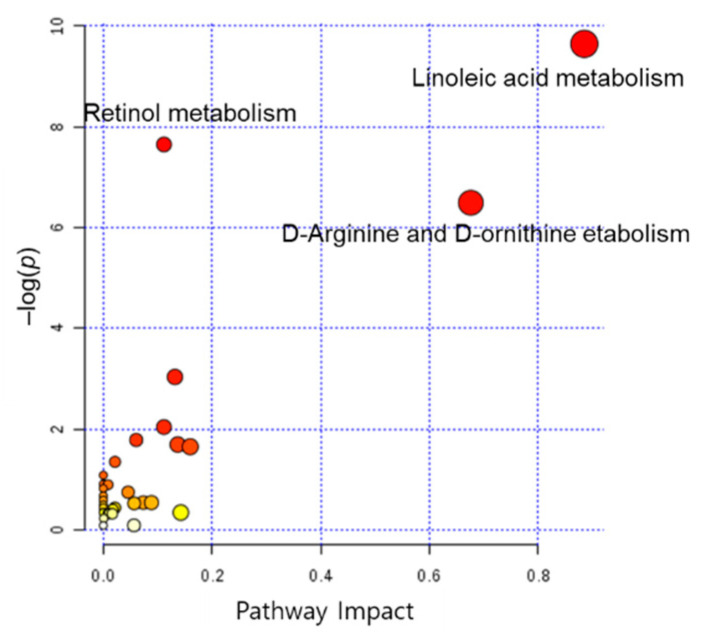
Scatter plot of pathway impact analysis. *y*-axis represents *p* value.

To better understand how metabolome changes in the maternal plasma are associated with PTB, we performed a metabolic pathway analysis. The pathway impact analysis was based on the KEGG database and MetaboAnalyst 3.0 for comprehensive metabolic data analysis, visualization, and interpretation [33,34]. Results of the impact pathway analysis are shown in Figure 3. The *x*- and *y*-axis represent the pathway topology analysis and pathway enrichment analysis, respectively. Metabolic pathways, including retinol, linoleic acid, and d-arginine and d-ornithine metabolism were shown to be associated with PTB.

### 2.4. Analysis of Targeted Plasma Metabolite

**Figure 4:**
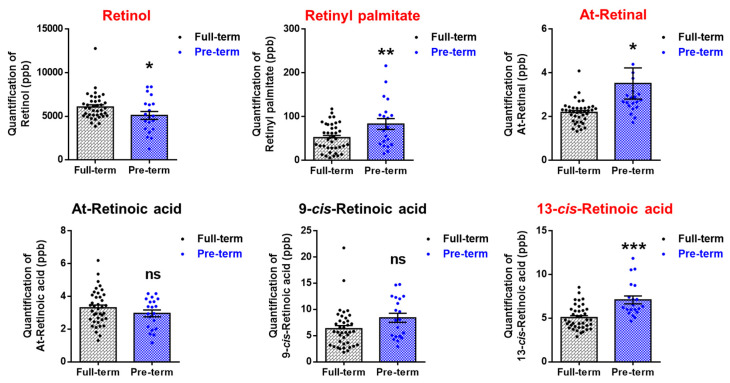
Retinoid metabolites showing changes in plasma of preterm birth using retinoid metabolite analysis (39 TB, 20 PTB). At: all trans. Student’s *t-*test, * *p* < 0.05, ** *p* < 0.01, *** *p* < 0.001, and ns = non-significant.

We next focused on the metabolites involved in retinoid metabolism to investigate the pathological mechanisms underlying PTB and to identify indicators for predicting PTB. The retinoid metabolites in the plasma of pregnant women were analyzed using LC-MS/MS (TB, *n* = 39; PTB, *n* = 20). When the gestational age was adjusted, remarkable differences were found in the amount of various retinol metabolites between PTB and TB (Figure 4 and Table S4). While retinol levels were significantly lower, retinal palmitate, all trans (At)-retinal, and 13-cis-retinoic acid (^13^cis-RA) levels were significantly higher in PTB than in TB (*p* < 0.05).

### 2.5. Analysis of Plasma RBP

We further analyzed the concentration of retinol-binding protein (RBP) to determine its correlation with retinol levels in a subset of maternal plasma. Although retinol was significantly decreased in PTB compared to TB, RBP levels were significantly increased in PTB (Table 2).

### 2.6. Predictive Performance for PTB

**Figure 5:**
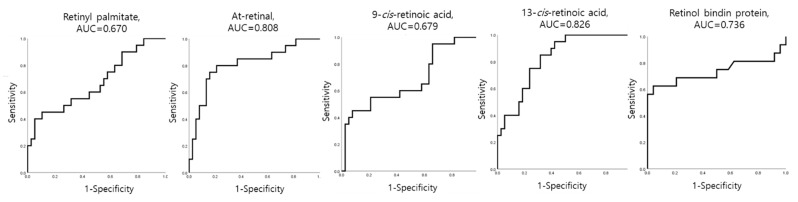
Receiver operating characteristics (ROC) curve analysis for metabolites: Predictive performance of signatures for preterm birth using ROC curves of sensitivity and specificity.

The diagnostic accuracy of PTB was summarized by evaluating the area under the curve (AUC) of the receiver operating characteristic (ROC) curves. All retinoid metabolites and RBP showed a significantly high predictive value (*p* < 0.01) of over 0.6 for PTB (Table 3, Figure 5). Among the four metabolites, At-retinal (AUC 0.808, 95% CI: 0.683–0.933) and ^13^cis-RA (AUC 0.826, 95% CI: 0.723–0.930) served as improved prediction markers for PTB. Interestingly, RBP levels were positively correlated with At-retinal (r = 0.415, *p* = 0.008) and ^13^cis-RA (r = 0.462, *p* = 0.003).

## 3. Discussion

We evaluated the plasma metabolome profiles and metabolic pathways to identify the etiological mechanism of PTB and indicators to predict PTB in pregnant women. Our results showed that the metabolome profile of PTB was significantly different from that of TB, especially At-retinal and ^13^cis-RA, which served as markers to significantly improve the prediction of PTB among the analyzed retinol metabolites. Although retinol levels were decreased in PTB, the RBP levels increased, showing a positive correlation with At-retinal and ^13^cis-RA. This is the first study to demonstrate the possibility of discovering indicators of PTB using plasma metabolites in pregnant Korean women.

Although the mechanisms of PTB and normal parturition are not well understood, the rupture of the fetal membranes is a common feature of TB and PTB. Understanding the membrane rupture process could provide important clues to premature rupture of membranes (PROM) in PTB [30]; pPROM accounts for one-third of spontaneous PTBs [35] and is strongly associated with adverse pregnancy outcomes [36,37]. Membrane rupture can result from biochemical changes in collagen structure and formation as well as increased oxidative stress [15,16]. Thus, classification of TB or PTB groups by metabolites as indicators may enable identification of high-risk pregnancies, especially with maternal peripheral blood as a useful biological sample for non-invasive biomarker identification [38,39].

A study reported that maternal plasma metabolites, in which plasma was collected directly after admission to the hospital, before steroid or tocolytic therapy, are different between TB and PTB parturitions [38]. Another study reported that serum retinol levels are essentially constant during pregnancy and lower in pregnant women and parturient mothers than in non-pregnant women [31]. We collected maternal blood at the time of an outpatient visit in the 2nd trimester or at delivery in our study. Despite the adjusted gestational age, plasma metabolome profiles were different in PTB compared with TB, with changes in retinoid metabolites associated with PTB. We previously reported that microbial composition differs between the blood of TB and PTB, and plasma in PTB is rich in *Butyricicoccus* and *Ruminococcaceae* belonging to *Clostridia* order [26]. Furthermore, production of vitamin A metabolites could be regulated by bacteria such as *Clostridia* in the gut [40,41]. This microbial imbalance suggests involvement in PTB by altering the production of vitamin A metabolites.

Vitamin A and its synthetic derivatives (retinoids) are crucial micronutrients for pregnant women and their fetuses [29]. In normal physiological levels, retinoids are essential for numerous biological functions such as cellular homeostasis, embryonic development, vision, tissue differentiation, growth, and mucus secretion [30]. Vitamin A is mainly dietary-derived fat-soluble signaling molecules and is stored about 80% of vitamin A (retinyl ester) in the stellate cells of the liver [30]. Pregnancy physiologically represents an inflammatory process in view of the immunological adaptations necessary to ensure the viability of the conceptus [42,43]. These physiological changes can alter the relationship between hepatic reserves and circulating retinol during pregnancy. Our results showed that levels of liver enzymes in the 3rd trimester of pregnancy did not differ between the plasma of TB and PTB, but C-reactive protein (CRP) levels were increased in PTB compared to TB. These results suggest that retinoid metabolites were associated with inflammation rather than liver enzymes.

Circulating retinol is transported in the plasma in a 1:1 complex with RBP [29], which has been proposed to play an important role in the delivery of retinoid from mother to fetus [44,45]. In this study, retinol levels were similar in TB and PTB plasma in the 2nd trimester, but decreased in PTB compared to TB in the 3rd trimester (Figure S1). Low plasma retinol can be caused by insufficient intake of vitamin A or inflammation [32]. However, considering that the study subjects were pregnant women, the significant decrease in retinol levels in the 3rd trimester can be considered that the process of delivery due to inflammation or infection occurs rapidly, leading to PTB.

RBP levels as well as At-retinal and ^13^cis-RA levels increased in PTB plasma in both 2nd and 3rd trimesters (Figure S1), with these metabolites positively correlated. RBP can be combined with retinol, At-retinal, and ^13^cis-RA and can be delivered to target tissues, such as the placenta and retina, through the Stra6 receptor [46,47]. In these tissues, vitamin A is then stored in the form of retinyl esters (mainly at the level of the liver) and is converted by the enzymatic cascade to the active forms AtRA and 9-cis-retinoic acid (^9^cis-RA) [31]. High levels of certain metabolites of retinoic acid (AtRA and ^13^cis-RA) can influence gene activity during critical periods of organogenesis and embryogenesis, leading to teratogenicity [30].

At gene level, retinoic acids bind to two effectors, retinoic acid receptor (RAR) and retinoic X receptor (RXR), which belong to a large family of nuclear receptors. They reside within the promoter of the gene and allow the regulation of transcription by vitamin A [29,46]. RAR and RXR may also be involved in pPROM by regulating the tissue-type plasminogen activator (PLAT) gene, which affects collagenolytic actions [48].

Taken together, this study revealed changes in metabolome profiles and target metabolites between the maternal plasma of TB and PTB. Particularly, At-retinal and ^13^cis-RA showed improved predictions for PTB, and At-retinal and ^13^cis-RA along with retinol was potentially bound to RBP and migrated to the target tissue, suggesting the possibility of involvement in amniotic membrane rupture. By employing a nested, case-control study design to match a discovery cohort of PTB cases with TB controls, we were able to increase statistical efficiency by having all cases and controls derived from the same well-defined source population, and to address relative confounders [49].

This study suggests that retinoid metabolism significantly improves the accuracy of PTB prediction and plays an important role in maintaining pregnancy and inducing early parturition. Further studies are required to confirm the relationship between PTB and retinoid metabolites in large groups amongst different races and to trace changes in retinoid metabolites among pregnant women during their pregnancies.

## 4. Materials and Methods

### 4.1. Study Subjects

The study was conducted according to the guidelines of the Declaration of Helsinki, and approved by the Institutional Review Board of Ewha Womans University Medical Center (EUMC 2018-07-007-010). Informed consent was obtained from all subjects involved in the study.

We recruited pregnant women who visited the Ewha Womans University Medical Center for prenatal examinations and delivery. These subjects were divided into a discovery group (10 TB and 11 PTB) to analyze the metabolite profile, and validation group (39 TB and 20 PTB) to analyze the retinoid metabolite. We collected maternal blood at the time of an outpatient visit in the 2nd trimester or at delivery in our study. The study subjects were recruited at the time of an outpatient visit in the 2nd trimester or at the hospitalization of singleton pregnant women with labor and/or premature rupture of membrane (RPOM) between 16 weeks and 40 weeks and 5 days of gestational age. Preterm labor (PTL) was diagnosed in patients with regular uterine contraction and 4 or more contractions in 20 min, or 8 or more in 60 min, as detected by cardiotocography. To diagnose preterm PROM, we conducted a sterile speculum examination to detect amniotic fluid pooling in the vaginal cavity, and a nitrazine test for detecting rupture of the membranes. Gestational age was determined on the first day of the last menstrual period and ultrasound examination. When pregnant women were visited for prenatal examinations or admitted to the hospital for delivery, blood samples were collected and stored at −80 °C until metabolomic analysis.

### 4.2. Maternal Blood Sample Preparation

Plasma samples (200 μL) were precipitated with 600 μL solvent mixture (acetonitrile/methanol/acetone; 1:1:1, *v*/*v*), mixed by vortexing for 3 min, then kept at −20 °C for 30 min. Precipitated samples were centrifuged at 10,000× *g* at 4 °C for 10 min, after which 600 μL of the supernatant was transferred into another test tube and evaporated to dryness under a gentle nitrogen stream. The residue was reconstituted with 200 μL of 50% methanol, and 5 μL (RPC separation mode) of samples was analyzed by UPLC-LTQ–Orbitrap MS. A pooled quality control (QC) sample was made by mixing equal amounts of samples and blank solvent used for checking system stability and sample carryover.

### 4.3. Metabolomic Profiling by LTQ-Orbitrap MS

Metabolite profiling was performed on an Ultimate 3000 UHPLC system (Thermo Fisher Scientific, San Jose, CA, USA) coupled with an LTQ-OrbitrapVelos Pro hybrid mass spectrometer (Thermo Fisher Scientific) equipped with an electrospray source operating in both positive mode (ESI+) and negative mode (ESI−). MS operation parameters were as follows: spray voltage, 3.5–5 kV; sheath gas, 5–45 (arbitrary units); auxiliary gas, 1 (arbitrary units); sweep gas, 1 (arbitrary units); and capillary temperature, 320 °C. Each sample was analyzed in FTMS full scan mode at a resolving power of 100,000 and *m*/*z* ranges were set to 50–1200 in centroid mode. The system was controlled by Xcalibur software v2.2, Tune Plus 2.7, and Chromeleon MS Link software v6.80 from Thermo Fisher Scientific.

Reversed-phase separation was performed on an Acquity™ UPLC BEH C18 column (2.1 mm × 100 mm, 1.7 μm, Waters, Milford, MA, USA) UPLC analytical column. The mobile phase solvents were 95% water, 5% ACN, 0.1% formic acid (mobile phase A), and 95% ACN, 5% water, and 0.1% formic acid (mobile phase B). The elution gradient was as follows: 100% mobile phase A from 0 to 3 min; linear increase to 50% mobile phase B from 3 to 10 min; linear increase of mobile phase B from 50% to 90% from 10 to 12 min; linear increase in mobile phase A from 100% to 12 to 15 min; and re-equilibration with 100% mobile phase A from 15 to 18 min. The column was maintained at 40 °C and the total run time was 18 min. A 5 μL aliquot of each sample was injected for analysis. The samples were stored at 4 °C using an auto sampler during the analysis.

The data processing procedure was as follows: Raw data were analyzed by Thermo Scientific SIEVE software v2.1 with “Small molecule”, “Chromatographic Alignment and Framing”, and “Nondifferential single class analysis” options. All data was scaled by the Pareto (Par) scaling method before analysis. Multivariate analysis (MVA) was performed using SIMCA-P software v14.0+ from Umetrics (Umea, Sweden) for partial least-squares discriminant analysis (PLS-DA). The variable importance in projection (VIP) value > 1 was considered significant. Statistical significance was analyzed with SPSS 22.0 (SPSS Inc., Chicago, IL, USA). The Mann-Whitney U-test was performed for a comparison of the two groups. Differences with *p*-value < 0.05 were considered statistically significant. Fold change was measured using the variation of the measured value. Pathway impact analysis was performed by Metaboanalyst 3.0 (Montreal, QC, Canada), a web-based metabolomics data processing tool and visualization metabolomics.

### 4.4. Preperation of Standard Stock Solution for Verification of Retinoid Metabolites

Retinoid metabolites were analyzed to establish the metabolic pathway differences between TB and PTB. Stock solutions of all indicated analytical reference and internal standards of the retinoids were uniformly dissolved in methanol to obtain final concentrations of 2000 ppm for retinol, retinyl acetate (RAc), retinyl palmitate (RP), all-trans retinal, At-RA, ^13^cis-RA, retinyl acetate-D6 (RAc-D6), all-trans retinal-D6 (At-RAL-D6), and all-trans retinoic acid-D6 (At-RA-D6). Retinol-D6 (ROH-D6) was prepared at a final concentration of 1000 ppm. Solutions were stored at −20 °C until use. Calibration curves for the final quantification of the target retinoids were generated by performing four-fold serial dilutions of these standard stock solutions in the range of 800 to 0.05 ppb. ROH, RAc, at-RAL, and at-RA standards were obtained from Cayman Chemicals, Inc. (Ann Arbor, MI, USA), and RP and ^13^cis-RA were acquired from Sigma Aldrich Corp. (St. Louis, MO, USA). All four isotope-labeled internal standards mentioned above were purchased from Cambridge Isotopes Laboratories, Inc. (Tewksbury, MA, USA).

### 4.5. Sample Preperation

A liquid-liquid extraction method was applied to extract the target retinoids from plasma samples. An isotope-labeled internal standard mixture was added to 200 μL of each plasma sample and vortexed briefly prior to serum protein precipitation with 200 μL acetonitrile. After vortexing for 1 min, 1.2 mL of methyl-tert-butyl ether was added to each tube and vortexed for 1 min. Samples were centrifuged at 13,000 rpm at 4 °C for 10 min, and the upper organic layer was transferred to a new test tube. The transferred supernatant was dried with nitrogen gas at room temperature, and the residue was reconstituted with 20 μL of methanol. After vortexing for 5 min, the supernatant was transferred to a clean glass MS vial tube and capped for LC-MS/MS analysis.

### 4.6. LC-MS/MS Analysis

Chromatographic separation of plasma sample extracts was performed on an Accucore C18 column (2.1 × 100 mm, 2.6 µm particle size, Thermo Scientific, Waltham, MA, USA), with the temperature maintained at 30 °C throughout the experiment. The injection volume of the samples was fixed at 2 μL. Target retinoids were separated within 22 min under a mobile phase composition consisting of 80% acetonitrile (A) and 100% methanol (B), both modified with 0.1% formic acid. The mobile phase system was initially maintained in isocratic mode at 100% of phase A for 7 min, with the flow rate linearly increased from 0.2 to 0.4 mL/min; it was then immediately changed to 100% of phase B and held for up to 15 min, with the flow rate maintained at 0.4 mL/min. For an additional 2 min, the system was gradually returned to 100% phase A with a linear gradient, and the flow rate was decreased back to 0.2 mL/min. Conditions were maintained for 5 min for re-equilibration. The column effluent was analyzed using an Agilent 6460C triple quadrupole LC-MS/MS system equipped with an electrospray ionization (ESI) source at Drug Development Research Core Center. Mass spectrometer was operated in positive ion mode using nitrogen as the nebulization gas. The temperature of the heated nebulizer was set to 300 °C with an ionspray voltage of 4500. Quantification of the retinoids was performed in multiple reaction monitoring (MRM) mode by selecting precursor ions of [M+H-fatty acid-H_2_O]^+^ for ROH, ROH-D6, RAc, RAc-D6, and RP, and [M+H]^+^ for At-RAL, At-RAL-D6, At-RA, ^9^cis-RA, ^13^cis-RA, and At-RA-D6. The optimized MRM transitions, MS parameters, and LC retention times of all the mentioned analytes are summarized in Table S5. LC-MS/MS spectra obtained from the blood sample were represented in Figure S2. For the final quantification, each peak area of the target retinoids was normalized to the response of corresponding internal standards (0.5 ppm ROH-D6 for ROH; 0.25 ppm RAc-D6 for RAc and RP; 0.25 ppm At-RAL-D6 for At-RAL; 1 ppm At-RA-D6 for At-RA, ^9^cis-RA, ^13^cis-RA). Overall quantitative analysis was performed using Agilent Mass Hunter software (Santa Clara, CA, USA).

### 4.7. Plasma RBP Analysis

Plasma RBP was analyzed using a Human RBP-ELISA kit (cat no. LS-F28830, Life span BioSciences Inc., Seattle, WA, USA). The assay procedure and reagent preparation were performed according to the manufacturer’s protocol.

### 4.8. Statistical Analysis

The basic characteristics of the study groups were compared using the Student’s t-test for continuous variables and the chi-square test for categorical variables. Multivariate analysis was performed to obtain a list of variables from the varied metabolic profiles in the plasma of pregnant women with TB and PTB. Analysis of covariance was performed to compare the levels of retinoid metabolites, by adjusting gestational age at sampling time. Multivariate analysis was performed using the SIMCA-P software v14.0+ (Umetrics, Umeå, Sweden). Pathway impact analysis and heat map visualization were performed using Metaboanalyst 3.0 (Montréal, QC, Canada), a web-based metabolomics data processing tool, and visualization metabolomics. Pathway mapping and chemical similarity analysis were performed using R version 3.2.2, MetaMapp, and CytoScape 3.4.0 (Boston, MA, USA) [50,51]. For verification using LC-MS/MS, differential metabolite levels were analyzed with a generalized linear model adjusted for gestational age at sampling time. The diagnostic accuracy of PTB was summarized by applying the AUC of the ROC curves. Statistical significance was set at *p* < 0.05. The Statistical Package for Social Sciences (SPSS, Version 2.0 Chicago, IL, USA) and online MEDCALC software were used for statistical analysis.

## Figures and Tables

**Table 1:** Clinical characteristics of subjects for validation (*n* = 59).

Characteristics	Term Birth (*n* = 39)	Preterm Birth (*n* = 20)	*p*-Value
Maternal age	33.3 ± 0.7	32.7 ± 1.0	0.626
GAS	23.0 ± 0.8	27.6 ± 1.6	0.010 *
preBMI	21.0 ± 0.4	21.7 ± 0.7	0.397
Parity			0.488
Nulliparous	16 (42.1)	8 (40.0)	
Multiparous	21 (57.9)	12 (60.0)	
Gravidity			0.416
0	25 (64.1)	11 (55.0)	
≥1	14 (35.9)	9 (45.0)	
WBC (×10^3^/mL)	8.8 ± 0.4	11.6 ± 0.6	<0.001 *
C-reactive protein (mg/dL)	0.3 ± 0.1	0.8 ± 0.2	0.034 *
pregBMI	26.8 ± 0.6	26.4 ± 0.8	0.397
GAD	39.0 ± 0.2	33.3 ± 1.0	<0.001 *
Delivery mode			0.026 ^†^
Normal delivery	23 (59.0)	5 (25.0)	
Cesarean section	16 (41.0)	15 (75.0)	
Birth weight (Kg)	3.2 ± 0.1	2.2 ± 0.2	<0.001 *
Gender, *n* (%)			0.651
Male	25 (64.1)	14 (70.0)	
Female	14 (35.9)	6 (30.0)	
APGAR 1 min	9.7 ± 0.1	7.9 ± 0.5	0.002 *
APGAR 5 min	9.9 ± 0.1	8.9 ± 0.4	0.009 *

Continuous variables are presented as mean ± SE. * Student’s *t*-test, *p* < 0.05; ^†^ χ^2^ test, *p* < 0.05. GAS, gestational age at sampling; preBMI, body mass index before pregnancy; pregBMI, body mass index at delivery; WBC, white blood cell; GAD, gestational age at delivery; APGAR, appearance, pulse, grimace, activity, respiration.

**Table 2:** Comparison of RBP levels between maternal blood of term and preterm birth.

Term Birth (*n* = 24, mg/L)	Preterm Birth (*n* = 16, mg/L)	*p-*Value
63.4 ± 4.2	122.9 ± 16.7 *	0.012

Data are presented as the mean ± SE. * Mann-Whitney *t*-test, *p* < 0.05.

**Table 3:** Efficacy of retinol-related metabolites for predicting preterm birth.

Metabolite	AUC	*p*-Value	SENS	SPEC	PPV	NPV	Accuracy
At-retinal	0.808	<0.001	75.0%	84.2%	68.2%	86.5%	79.7%
13cis-RA	0.826	<0.001	85.0%	68.4%	58.6%	90.0%	74.6%
9cis-RA	0.679	0.026	45.0%	92.1%	69.2%	76.1%	74.6%
Retinyl palmitate	0.670	0.035	40.0%	94.7%	72.7%	75.0%	74.6%
RBP	0.736	0.012	62.5%	95.8%	90.9%	79.3%	82.5%

Receiver operating characteristic (ROC) curve analysis was performed for statistical analysis, and *p* < 0.05, was considered significant. AUC, area under the curve; SENS, sensitivity; SPEC, specificity; PPV, positive predictive value; NPV, negative predictive value; OR, odds ratio; At, all trans; RA, retinoic acid; RBP, retinol binding protein.

## Data Availability

The data presented in this study are available from the corresponding author, Y.K., upon reasonable request.

## Supplementary Materials

The following are available online at https://www.mdpi.com/article/10.3390/metabo11070443/s1, Table S1: Clinical characteristics of subjects for analysis of untargeted metabolome profiles, Table S2: Comparison of blood indices related retinoid metabolism between TB and PTB (*n* = 59), Table S3: Ranked results of multivariate analysis on variable ions identified by UPLC-LTQ–Orbitrap MS, Table S4: Comparison of retinoid levels adjusted gestational age between maternal plasma of term and preterm birth, Table S5: LC retention times, MRM transitions, and MS parameters of target analytes, Figure S1: Changes in retinoid metabolites between 2nd and 3rd trimester.
