# Supplementary material for: Identification of Indicators for Preterm Birth Using Retinoid Metabolites
Source: Metabolites. 2021 Jul 7;11(7):443. doi: 10.3390/metabo11070443 (PMC8304766; doi:10.3390/metabo11070443)
Supplement: Supplementary file 1 [file metabolites-11-00443-s001.zip › metabolites-1262739-supplementary.pdf]

Article

Identification of potential biomarker of PTB using retinoid metabolites

Young-Ah You [1]†, Soo-Yeon Hwang[2]†, Soo Min Kim [1], Seojeong Park[2], Ga In Lee[3], Sunwha Park[1], AbuZar Ansari[1], Jeongae Lee[4], Youngjoo Kwon[2]* and Young Ju Kim [1,3]*

1 Department of Obstetrics and Gynecology, College of Medicine and Ewha Medical Institute, Ewha Womans University, Seoul 07804, Republic of Korea; yerang02@naver.com (Y.-A.Y.), zeus_0218@naver.com (S.M.K.), loveleee0102@gmail.com (G.I.L.) clarrissa@hanmail.net (S.P.), abu.zar.0313@outlook.com (A.A.),

2 Department of Pharmacy, Ewha Womans University, Seoul 03760, Republic of Korea

3 Graduate Program in System Health Science and Engineering, Ewha Womans University, Seoul, Republic of Korea

4 MolecularRecognition Research Center, Korea Institute of Science and Technology, Seoul 02792, Korea

* correspondence author: ykwon@ewha.ac.kr (Y.K.), kkyj@ewha.ac.kr (Y.J.K.)

† Equal contribution as first author

Received: date; Accepted: date; Published: date

Table S1. Clinical characteristics of subjects for analysis of untargeted metabolome profiles (n=21).

	Term birth (*n* = 10) Median (IQR)	Preterm birth (*n* = 11) Median (IQR)	*p*-value
Maternal age	33.0 (4.8)	33.0 (4.8)	0.669
GAS	38.5 (1.6)	31.4 (7.4)	< 0.00*
Parity			0.423
Nulliparous, n	3	4	
Multiparous, n	7	7	
Gravidity			0.237
0, n	3	7	
≥ 1, n	7	4	
WBC (x 10^3/ml)	9.8 (2.9)	10.3 (8.4)	0.468
C-reactive protein	-	0.5 (0.9)	-
AST	21.0 (7.3)	21.0 (3.0)	0.809
ALT	14.5 (3.8)	14.0 (9.0)	1.000
AST/ALT ratio	1.4 (0.6)	1.4 (0.7)	0.918
TC	259.5 (29.0)	253.0 (36.0)	0.512
TG	259.0 (115.5)	178.0 (148.0)	0.114
BUN	7.0 (1.6)	6.0 (6.0)	0.863
Creatinine	0.7 (0.1)	0.6 (0.2)	0.223
BUN/Creatinine ratio	10.1 (2.9)	10.1 (8.8)	0.705
pregBMI	25.2 (3.3)	25.0 (3.7)	0.029*
GAD	38.5 (1.6)	33.0 (8.6)	< 0.001*
Delivery mode			0.063
Normal delivery, n	7	3	
Cesarean section, n	3	8	
Birth weight (Kg)	3.3 (0.3)	2.0 (1.5)	< 0.001*
Gender, n (%)			0.426
Male, n	7	7	
Female, n	3	4	
APGAR 1min	10.0 (0.0)	6.0 (6.0)	< 0.001*
APGAR 5min	10.0 (0.0)	9.0 (3.0)	0.003*

Data were presented as Median (Interquartile range, IQR). * Mann Whitney test, $p < 0.05$; †$\chi 2$ test, $p < 0.05$. GAS, gestational age at sampling; PregBMI, body mass index at delivery; GAD, gestational age at delivery.

Table S2. Comparison of blood indices related retinoid metabolism between TB and PTB (n=59).

	Term Birth (n=39)	Preterm birth (n=20)	*p*-value
AST	16.92 ± 0.70	17.75 ± 1.38	0.554
ALT	11.79 ± 1.17	12.95 ± 2.00	0.597
AST/ALT	1.69 ± 0.09	1.62 ± 0.11	0.642
TC	263.64 ± 7.07	257.75 ± 9.96	0.630
TG	307.23 ± 24.90	264.85 ± 29.62	0.303
BUN	8.41 ± 0.53	7.50 ± 0.77	0.328
Creatinine	0.57 ± 0.02	0.50 ± 0.03	0.066
BUN/Creatinine	14.75 ± 0.56	15.60 ± 1.08	0.441

Data were presented as Mean ± SE. Student's t-test, AST, aspartate aminotransaminase; ALT, alanine aminotransferase; TC, total cholesterol; TG, triglyceride; BUN, blood urea nitrogen

Table S3. Ranked results of multivariate analysis on variable ions identified by UPLC-LTQ–Orbitrap MS.

No.	RT_Mass	Detected method	VIP value	*p*-value	Change	Fold change(P/C)	Accurate Mass	Mass error(ppm)	Metabolic pathway
1	11.61_163.0094	RP	2.66	1.51E-03	↑	7.41			
2	11.36_319.2058	RP	2.41	3.60E-02	↑	1.94	319.2034	7.83	Linoleic acid metabolism
3	10.92_274.2816	RP	2.09	2.63E-02	↓	0.79			
4	5.96_90.5243	RP	2.05	2.74E-02	↑	6.51			
5	11.63_270.2154	RP	1.98	1.72E-02	↑	3.34	270.2342	69.57	Retinol metabolism
6	11.35_133.1127	RP	1.85	2.50E-02	↓	0.34	133.0972	116.46	Arginine and ornithine metabolism
7	11.58_121.102	RP	1.54	3.07E-02	↑	8.11			
8	10.97_106.0885	RP	1.53	4.25E-02	↓	0.73			
9	10.2_95.0765	RP	1.34	4.65E-02	↑	2.60			
10	11.53_834.6856	RP	1.30	3.95E-02	↑	61.76			
11	2.16_90.5248	RP	1.29	4.76E-02	-	-			
12	11.36_145.0167	RP	1.19	1.44E-02	↑	1.75			

13	10.99_334.3113	RP	1.16	3.45E-02	↓	0.61			
14	9.22_95.0809	RP	1.00	3.87E-02	↑	2.48			
15	11.42_117.0732	RP	0.97	4.47E-02	↑	9.68	117.0546	158.90	Arginine and ornithine metabolism
16	9.67_369.1706	RN	1.35	2.13E-02	↑	6.51	369.1838	35.75	Arachidonic acid metabolism
17	11.5_685.5345	RN	1.35	5.63E-03	↑	11.66			
18	0.82_313.0628	RN	0.98	4.40E-02	↑	2.20	313.1055	136.38	Folate biosynthesis

Table S4. Comparison of retinoid levels adjusted for gestational age between maternal plasma of term and preterm birth (TB, n = 39; PTB, n = 20).

Metabolites	ANCOVA	
	F	*p*-value
Retinyl palmitate	5.916	0.018
Retinol	0.818	0.370
At-Retinal	7.080	0.010
At-Retinoic acid	0.482	0.490
9-*cis*-Retinoic acid	4.015	0.050
13-*cis*-Retinoic acid	18.059	<0.0001

Statistical analysis were performed using analysis of covariance (ANCOVA).

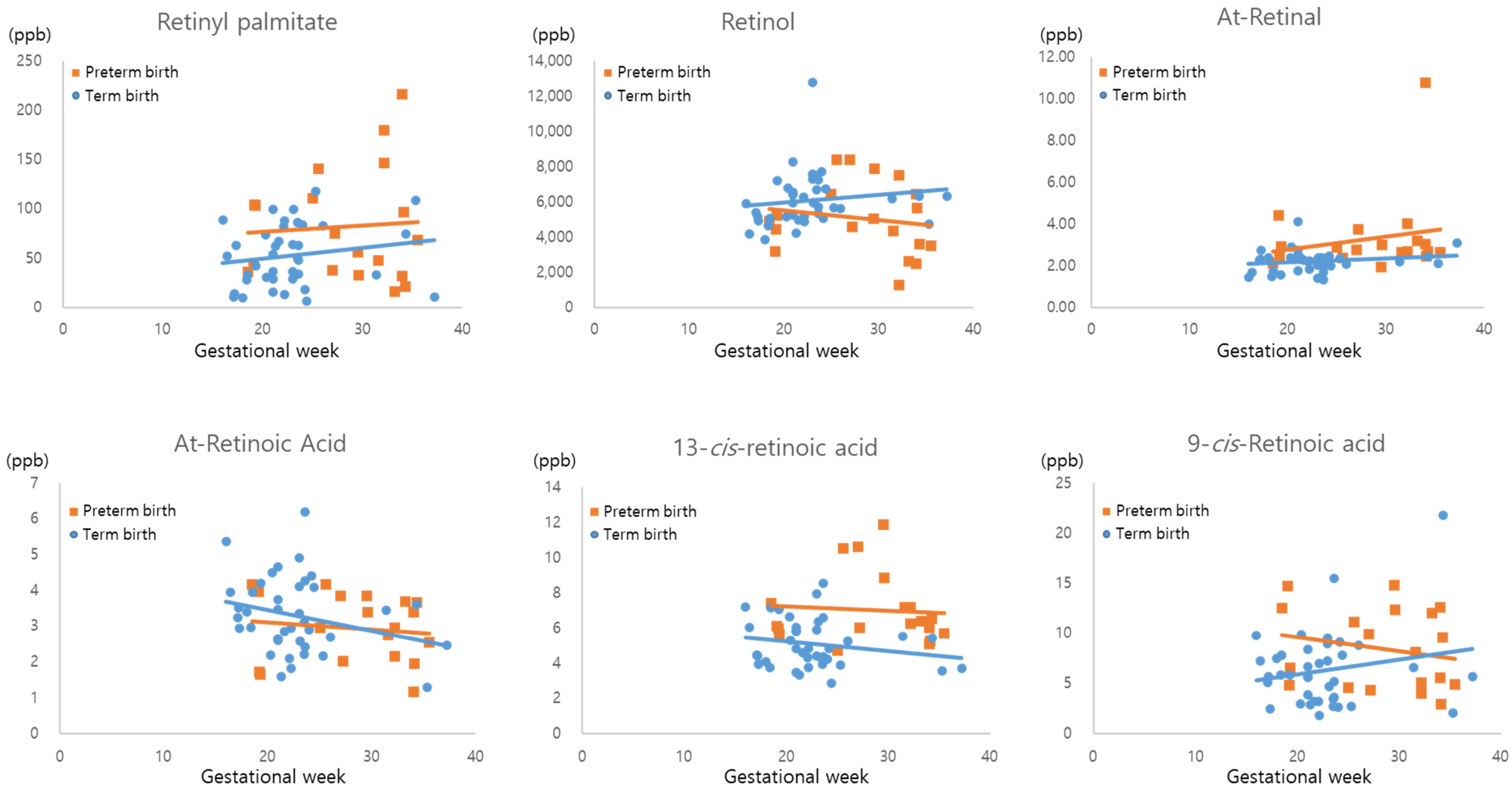

Figure S1. Changes in retinoid metabolites between the 2nd and 3rd trimester (TB, n=39; PTB, n=20). At : all trans.

Table S5. LC retention times, MRM transitions, and MS parameters of target analytes

**Analytes**	**R_t (min)**	**polarity**	**Precursor (m/z)**	**Product (m/z)**	**Fragmentor Energy (V)**	**Collision Energy (V)**
^{13}*Cis*-retinoic acid	3.128	positive	301.2	123.1	105	12
^{9}*Cis*-retinoic acid	3.334	positive	301.2	123.1	105	12
All-trans retinoic acid	3.530	positive	301.1	159.1	115	18
Retinoic acid-D6*	3.531	positive	307.2	165.2	115	18
All-trans retinal	4.679	positive	285.2	161.1	115	2
Retinal-D6*	4.679	positive	291.2	69	95	22
Retinol	3.674	positive	269.2	93.1	115	18
Retinol-D6*	3.674	positive	275.2	96	110	14
Retinyl acetate	6.889	positive	269.2	96	108	21
Retinyl acetate-D6*	6.889	positive	275.2	96	115	16
Retinyl palmitate	13.982	positive	269.1	96	111	21

* Internal standards

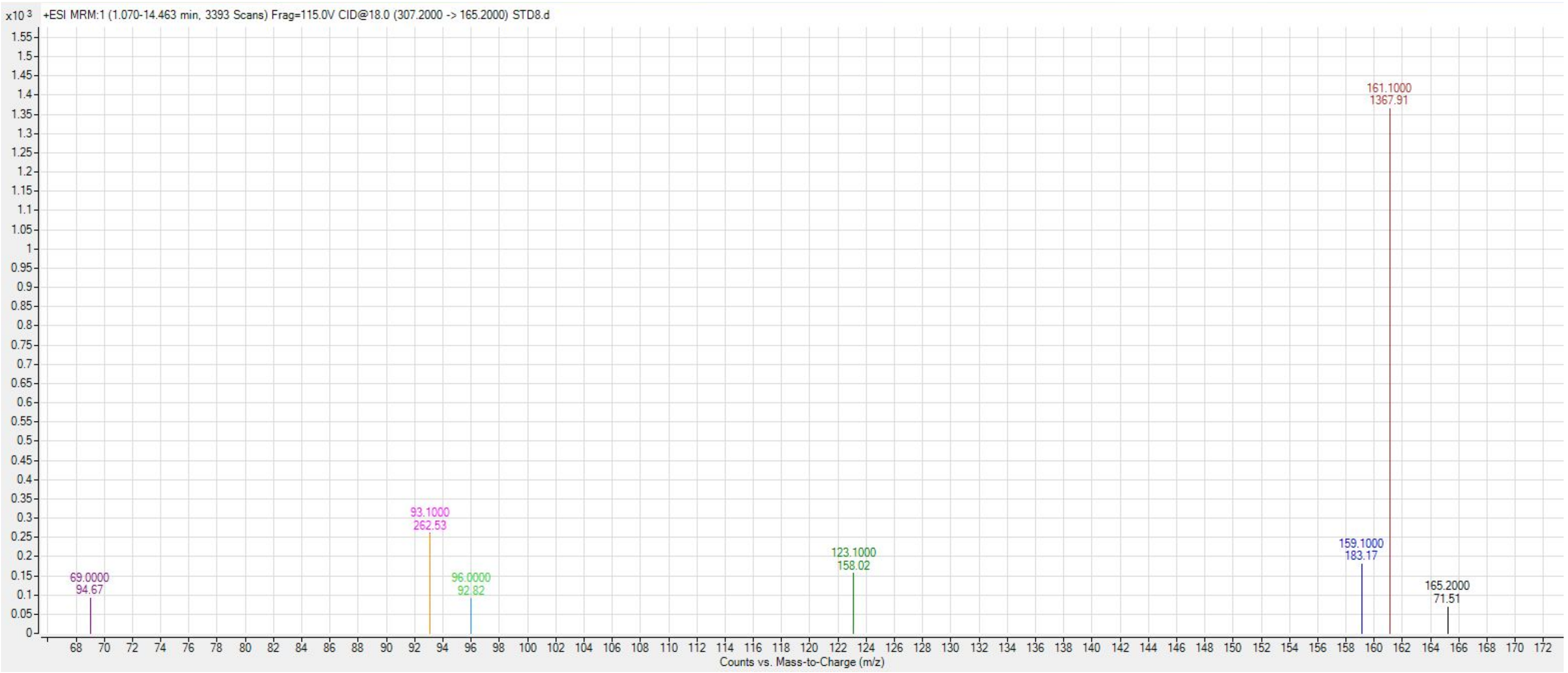

Figure S2. Representative LC-MS/MS spectra obtained from the blood sample.
